# Interplay of Vitamin D, Erythropoiesis, and the Renin-Angiotensin System

**DOI:** 10.1155/2015/145828

**Published:** 2015-04-27

**Authors:** Domenico Santoro, Daniela Caccamo, Silvia Lucisano, Michele Buemi, Katerina Sebekova, Daniel Teta, Luca De Nicola

**Affiliations:** ^1^Department of Clinical and Experimental Medicine, University of Messina, Via Faranda, 2-98123 Messina, Italy; ^2^Department of Biomedical Sciences and Morphological and Functional Imaging, University of Messina, Italy; ^3^Comenius University, Bratislava, Slovakia; ^4^University Hospital (CHUV), Lausanne, Switzerland; ^5^Second University of Naples, Naples, Italy

## Abstract

For many years deficiency of vitamin D was merely identified and assimilated to the presence of bone rickets. It is now clear that suboptimal vitamin D status may be correlated with several disorders and that the expression of 1-*α*-hydroxylase in tissues other than the kidney is widespread and of clinical relevance. Recently, evidence has been collected to suggest that, beyond the traditional involvement in mineral metabolism, vitamin D may interact with other kidney hormones such as renin and erythropoietin. This interaction would be responsible for some of the systemic and renal effects evoked for the therapy with vitamin D. The administration of analogues of vitamin D has been associated with an improvement of anaemia and reduction in ESA requirements. Moreover, vitamin D deficiency could contribute to an inappropriately activated or unsuppressed RAS, as a mechanism for progression of CKD and/or cardiovascular disease. Experimental data on the anti-RAS and anti-inflammatory effects treatment with active vitamin D analogues suggest a therapeutic option particularly in proteinuric CKD patients. This option should be considered for those subjects that are intolerant to anti-RAS agents or, as add-on therapy, in those already treated with anti-RAS but not reaching the safe threshold level of proteinuria.

## 1. Introduction

The kidney has an important role in the regulation of several systems. In addition to excretory activity, regulation of water and electrolytes, and maintaining normal acid-base homeostasis, the kidney has also an endocrine function. It is carried out through the production of important hormones: renin, prostaglandins, erythropoietin, and calcitriol.

Renin is released from the renal juxtaglomerular apparatus (JGA). Renin production is regulated by three major mechanisms: change in renal perfusion pressure, solute delivery to the macula densa cells, and influence of renal sympathetic nerves. The negative effects of the activation of renin-angiotensin system on the progression of renal failure are well known. Indeed, blockade of the renin-angiotensin system is a widely established and utilized antiproteinuric and renoprotective modality [1, 2].

**Figure 1:**
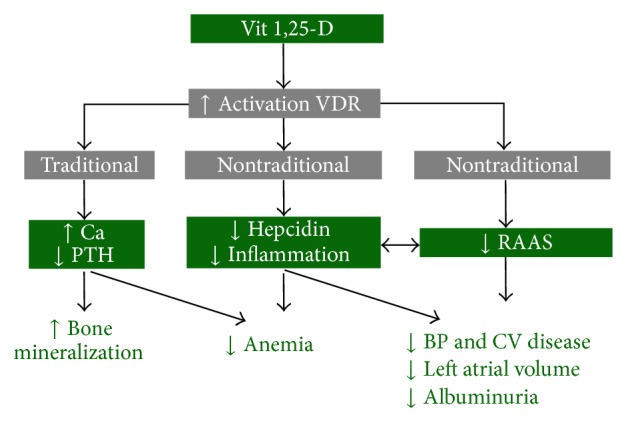
Traditional and nontraditional effects of active vitamin D.

Moreover, it has been suggested that the dual-block therapy might improve outcome by preventing compensatory feedback processes that generate more angiotensin II when a single blocker is used. Indeed a number of studies on the progression of renal disease were focused on the role of blocking the activation of the renin-angiotensin system (RAS), to reduce the loss of glomerular filtration rate and delay the start of dialysis, even though the results on the safety of this intervention do suggest caution on the “aggressive” suppression of RAS [3, 4]. However, a reduced efficacy of RAS inhibitors is due to the compensatory increase of renin synthesis caused by the disruption of the feedback inhibition loop. Renin build-up in fact not only stimulates the conversion of Ang I leading to Ang II accumulation but is also likely associated with detrimental effects directly induced by renin [5]. In this context it is important to value the role of vitamin D. Experimental evidence in fact has accumulated on vitamin D-related blunting of the compensatory increase of renin synthesis occurring during chronic administration of anti-RAS agents [6, 7]. In particular, in experimental diabetes, block of the compensatory increase of renin expression by vitamin D analogs dramatically increases the therapeutic efficacy of RAS inhibition (Figure 1) [7]. Beyond the known effect on blood pressure recent studies provided valuable insight into the nonhemodynamic actions of Ang II and other components of the RAS in the progression of kidney disease [8].

In patients with chronic renal disease, a slow, gradual decrease in the level of 1,25-dihydroxyvitamin D (calcitriol) and erythropoietin is observed whilst different mechanisms bring to increased activation of the renin-angiotensin system [9]. The main complications of erythropoietin and 1,25-dihydroxyvitamin D (calcitriol) deficiency are in fact anemia and secondary hyperparathyroidism. Renal anemia is due to a reduced production of erythropoietin by interstitial fibroblast in the renal cortex, between tubular epithelial cells and peritubular capillaries [10]. The origin of decreased serum levels of 1,25(OH)^2^D is multifactorial. The leading cause is a decrease in renal mass, which causes a consequent reduction in the level of 1-*α*-hydroxylase available for the production of active vitamin D. In CKD, hyperparathyroidism and hyperphosphoremia can contribute to inhibit the renal bioactivation of vitamin D.

While single effects of the different renal hormones are well known, less information is available regarding the interaction between them. Recently, a number of studies suggested that vitamin D interplays with both renin-angiotensin system and erythropoietin [11, 12]. This interaction would be responsible for some of the systemic and renal effects which has recently been implicated for vitamin D. In particular, the interplay between renal hormones produces its effects on hypertension and proteinuria [13].

## 2. Vitamin D and Erythropoietin

Recent clinical observations suggest a possible role of vitamin D in erythropoiesis [14]. In the hemodialysis population, 1-25(OH)D repletion has been associated with dose reductions in erythrocyte-stimulating agents (ESA) and increased reticulocytosis [15, 16].

In CKD patients, the administration of either nutritional or active vitamin D has been associated with an improvement of anaemia and reduction in ESA requirements [17].

Despite these intriguing observations, there is overall paucity of clinical studies investigating whether adequacy of 1-25(OH)D affects blood hemoglobin (Hb) levels. Patel et al. show that 25D and 1,25D deficiency are independently associated with decreased hemoglobin levels and anemia in chronic kidney disease. They measured the concentrations of 25-hydroxyvitamin D (25D), 1,25-dihydroxyvitamin D (1,25D), and hemoglobin in a cross-sectional study of 1661 subjects in SEEK, a multicenter cohort study of chronic kidney disease patients in the United States, of whom 41% met the criteria for anemia. The mean hemoglobin concentrations significantly decreased with decreasing tertiles of 25D and 1,25D. These linear trends remained significant after adjustment for age, gender, ethnicity, eGFR, diabetes, and PTH [12].

To evaluate the prevalence of anemia in a population of individuals with vitamin D deficiency, Sim et al. studied for two years 554 subjects in a general population as part of normal healthcare operations. Anemia was present in 49% of 25(OH)D-deficient subjects compared with 36% with normal 25(OH)D levels (*P* < 0.01). 25(OH)D-deficient subjects had a lower mean Hb levels (11.0 versus 11.7; *P* = 0.12) and a higher prevalence of ESA use (47% versus 24%; *P* < 0.05). This study demonstrates an association between vitamin D deficiency, greater risk of anemia, lower mean hemoglobin, and higher use of ESA [18].

In end-stage heart failure subjects, vitamin D deficiency has been showed to be independently associated with low Hb values and anemia. In these subjects, the mean Hb concentrations were significantly reduced in the lower tertiles of 25(OH)D and 1,25(OH)^2^D (*P* < 0.001). The odds ratios for anemia of the lowest tertile of 25(OH)D (<18 nmol/L) and 1,25(OH)^2^D (<40 pmol/L) were 2.69 (1.46–5.00) and 4.08 (2.18–7.62) compared with their respective highest tertile (>32 nmol/L and >70 pmol/L). Patients with severe dual deficiency of 25(OH)D and 1,25(OH)^2^D had an odds ratio for anemia of 9.87 (95% CI 3.59–27.1) compared with patients in the highest tertile for both vitamin D metabolites [19].

Although vitamin D appears to be associated with anemia, the mechanism is unknown.

A reverse correlation was found between PTH and Hb level [20]. Possible causes of low Hb level or anemia due to SHPT may be because of increased bone marrow fibrosis, which may lead to decreased erythropoietin and increased resistance to EPO [21]. Erythropoietin cells express calcitriol receptors, which induces proliferation and maturation of erythroid progenitor cells. Therefore, deficiency of calcitriol, a cause of hyperparathyroidism, may impair erythropoiesis (Figure 1). There are also some studies, which support an increase in erythrocyte osmotic fragility due to high concentration of PTH in patients on dialysis, leading to low Hb level [22]. There is also indirect evidence of restoration of the hematocrit after parathyroidectomy in uremic patients due to restoration of bone marrow space after operation and rise of immunoreactive erythropoietin (EPO) serum concentrations [23]. Icardi et al. on the contrary consider that these effects are not related to parathyroid hormone (PTH) values and seem to be independent of PTH suppression [24].

The majority of studies concerning vitamin D deficiency or supplementation, and degree of renal anaemia, point out the prevalent role of inflammation in the mechanism underlying these associations. Immune cells express the vitamin D receptor (VDR) which in turn is involved in the modulation of innate and adaptive immunity. Both* in vivo* and* in vitro* studies have demonstrated that calcitriol reduces cytokines production [25]. VDR activation inhibits the expression of inflammatory cytokines in stromal and accessory cells and upregulates the lymphocytic release of interleukin-10 (IL-10) exerting both anti-inflammatory activity and proliferative effects on erythroid progenitors. In CKD patients, vitamin D deficiency may stimulate immune cells within the bone marrow microenvironment to produce cytokines, inducing impaired erythropoiesis. Immune activation involves the reticuloendothelial system, increasing hepcidin synthesis and functional iron deficiency [24]. Recently Zughaier et al. showed that 1,25-dihydroxyvitamin-D(3) (1,25(OH)^2^D3), the hormonally active form of vitamin D, is associated with decreased hepcidin and increased ferroportin expression in lipopolysaccharide (LPS) stimulated THP-1 cells. 1,25(OH)^2^D3 also resulted in a dose-dependent decrease in prohepcidin cytokines, IL-6, and IL-1*β*, release* in vitro*. Further, they show that high-dose vitamin D therapy impacts systemic hepcidin levels in subjects with early stage CKD. These data suggest that improvement in vitamin D status is associated with lower systemic concentrations of hepcidin in subjects with CKD [26].

Another possible explanation may be that calcitriol directly stimulates erythroid progenitors; vitamin D has been demonstrated to affect bone marrow function [27, 28].

Furthermore, levels of 1,25 hydroxyvitamin D (1-25(OH)^2^D), the active form of vitamin D, are several hundredfold higher in bone marrow compared with plasma [25]. Aucella et al. have shown that administration of 1,25(OH)^2^D increased burst-forming unit erythroid proliferation in patients with ESRD. Calcitriol has a direct effect on erythroid precursors proliferation, as demonstrated both* in vitro* and* in vivo*, with a synergistic effect with epoetin alfa [29]. Vitamin D receptors have been discovered in numerous nonrenal target tissues including the bone marrow [27, 28]. Normalizing tissue 25(OH)D levels may provide an adequate substrate for local tissue production of 1,25(OH)^2^D in hematopoietic tissues via extra-renal tissue activity of the 1-alpha-hydroxylase enzyme. Hematons, the buffy coat of bone marrow containing erythroid precursors, fibroblast, endothelial cells, lipid laden cells, and macrophages, have been demonstrated to contain significantly higher concentrations of 25(OH)D and 1,25(OH)^2^D levels than bone marrow plasma [16]. High local concentrations of 1,25(OH)^2^D in hematopoietic tissues may then directly activate erythroid precursor cells in a paracrine fashion.

In conclusion, only few studies with a limited number of patients explored the association between vitamin D deficiency and anemia in CKD patients. In addition, the molecular evidence about the role of calcitriol in erythropoiesis is still very limited.

## 3. Vitamin D and Renin-Angiotensin-Aldosterone System

The renin-angiotensin-aldosterone system (RAS) plays a central role in the regulation of blood pressure, electrolyte, and volume homeostasis. Epidemiological and clinical studies have repeatedly evidenced the impact of vitamin D on RAS activity at the clinical, pathophysiological, and molecular level.

RAS includes a cascade that leads to the generation of angiotensin II (Ang II), the main effector of the system. The rate-limiting component of the RAS is renin, a highly specific aspartic peptidase synthesized and secreted predominantly by the juxtaglomerular (JG) cells in the nephron. The only known substrate of renin is angiotensinogen, which is enzymatically cleaved to angiotensin I by renin. Angiotensin I is further cleaved to Ang II by the angiotensin converting enzyme (ACE) [11].

Ang II exerts diverse actions in multiple organs, including the brain, heart, kidney, adrenal glands, and peripheral vasculature, to regulate the blood pressure and electrolyte and extracellular volume balance and inappropriate stimulation of the RAS has been associated with hypertension, heart attack, and stroke [30, 31].

The relationship between vitamin D and blood pressure and/or plasma renin activity has been debated in many studies. The first clinical studies suggesting an inverse relationship between calcitriol and renin levels were published by Burgess, Resnick et al. more than two decades ago [32, 33]. This correlation was recently confirmed in a large cohort study of CKD patients by Forman et al. They examined the relation between plasma 25-hydroxyvitamin D and elements of the RAS in 184 normotensive individuals in high sodium balance; these included circulating levels of plasma renin activity and Ang II, and the renal plasma flow response to infused Ang II, which is an indirect measure of the intrinsic RAS activity in the kidney. Compared to individuals with sufficient 25-hydroxyvitamin D levels (≥30 ng/mL), those with insufficiency (15–29.9 ng/mL) and deficiency (<15 ng/mL) had higher circulating Ang II levels (*p*-trend = 0.03). Moreover, those with vitamin D deficiency had significantly blunted renal plasma flow responses to infused Ang II (mean decrease of 115 mL/min/1.73 m^2^ in renal plasma flow versus 145 mL/min/1.73 m^2^ among those with sufficient vitamin D levels; *P* value = 0.009). Although plasma renin activity was higher among individuals with insufficient levels of vitamin D, the result was not statistically significant. These data suggest that low plasma 25-hydroxyvitamin D levels may result in upregulation of the RAS in otherwise healthy humans [34, 35].

Furthermore Park et al. studied fifteen hemodialysis patients with secondary hyperparathyroidism. They showed that, in patients receiving calcitriol, levels of plasma renin (18.5/−12.7 v 12.3/−11.0 pg/mL; *P* = 0.007) and angiotensin II (AT N; 79.7/−48.6 v 47.2/−45.7 pg/mL; *P* = 0.001) were significantly decreased [36].

Several mechanistic studies confirming negative regulation of the renin gene by calcitriol have been published by the group of Li et al., who showed that renin expression and plasma angiotensin II production were increased severalfold in vitamin D receptor-null (VDR-null) mice, leading to hypertension, cardiac hypertrophy, and increased water intake. In wild-type mice, inhibition of 1,25-dihydroxyvitamin-D(3) synthesis also led to an increase in renin expression, whereas 1,25-dihydroxyvitamin-D(3) injection led to renin suppression [37]. In another study they demonstrated that suppression of renin expression by 1,25-dihydroxyvitamin D* in vivo* is independent of parathyroid hormone (PTH) and calcium [38]. To explore the molecular mechanism, they analyzed the mouse Ren-1c gene promoter by luciferase reporter assays. The data obtained indicate that calcitriol binds to the VDR and subsequently blocks formation of the cyclic adenosine monophosphate-response element-binding protein (CRECREB- CBP) complexes in the promoter region of the renin gene, reducing its level of expression [39].

Studies on suppression of renin-angiotensin gene expression in the kidney by paricalcitol were also conducted. Freundlich et al. studied rats with the remnant kidney model of chronic renal failure (5/6 nephrectomy) to which have been given two different doses of paricalcitol thrice weekly for 8 weeks. Paricalcitol was found to decrease angiotensinogen, renin, renin receptor, and vascular endothelial growth factor mRNA levels in the remnant kidney by 30–50 percent compared to untreated animals. Similarly, the protein expressions of renin, renin receptor, the Ang type 1 receptor, and vascular endothelial growth factor were all significantly decreased. Glomerular and tubulointerstitial damage, hypertension, proteinuria, and the deterioration of renal function resulting from renal ablation were all similarly and significantly improved with both treatment doses [40].

In a recent study Fryer et al. show that, in C57/BL6 mice administered vehicle, paricalcitol produces significant, dose-dependent suppression of renin expression in the absence of hypercalcemia at doses 10-fold above those necessary for PTH suppression. Calcitriol also produced suppression of renin at doses at least 10-fold above those required for PTH suppression, but increases in iCa(2+) were observed at doses only 3-fold above those necessary to elicit renin suppression.

Interactions between vitamin D and other system RAAS components have been studied as well [41].

Aldosterone binds mineralocorticoid receptor, which belongs to the same superfamily of nuclear receptors as the VDR. Therefore, cross talk between these receptors and their agonists could potentially exist. Fischer et al. observed that plasma concentration of 1,25-dihydroxyvitamin-D(3) and aldosterone were significantly higher in mice that are genetically deficient for klotho, a membrane protein participating in the inhibitory effect of fibroblast growth factor-23 (FGF23) on the formation of 1,25-dihydroxyvitamin-D(3). High levels of calcitriol were associated with hyperaldosteronism, which is similarly reversed by a vitamin D-deficient diet [42]. Furthermore Good et al. identified a novel regulatory interaction whereby aldosterone acts via nongenomic mechanisms to enhance the genomic response to 1,25-dihydroxyvitamin-D(3). Aldosterone may influence a broad range of biological processes, including epithelial transport, by modifying the response of target tissues to 1,25-dihydroxyvitamin-D(3) stimulation [43].

It has been demonstrated that low vitamin D status adversely affects cardiac function. This effect of vitamin D seems to be mediated by the renin-angiotensin system. Indeed, VDR-knockout mice show myocardial renin overexpression and marked cardiomyocyte hypertrophy [44].

Despite many studies suggested that vitamin D may favorably influence myocardial hypertrophy, two large randomized clinical trials have shown that VDR activation did not influence or reverse left ventricular hypertrophy [45, 46]. In particular in the PRIMO trial, which included 227 patients with CKD stages 3 to 4 who were randomized to paricalcitol or placebo, the change in left ventricular mass index after 12 months did not differ between the two groups [45]. Similar results were reported in the OPERA trial, where patients with 3 to 5 CKD were randomly assigned to receive oral paricalcitol or placebo. After 52 weeks, VDR activation with paricalcitol failed to demonstrate any change in the measures of LV structure and function. However in both studies the authors found a correlation between VDR activation and hospitalization for cardiovascular events [46]. Interestingly a post hoc analysis of PRIMO trial has demonstrated that forty-eight weeks of therapy with paricalcitol significantly reduces left atrial volume and attenuates the rise of brain natriuretic peptide (Figure 1) [47].

Chronic kidney disease (CKD) is a public health priority due to the prevalence rates, around 10% in general adult population, and the ominous and costly cardiorenal outcome [48]. Nowadays, albuminuria is widely considered the main “modifiable” risk factor of global prognosis in CKD patients. Even moderate increases of albuminuria in fact remarkably enhance the risk of end-stage renal disease (ESRD) and all-cause and cardiovascular (CV) death independently of age, hypertension, and diabetes [49, 50]. More important, a recent study in a cohort of 638,150 adults from a province-wide registry in Alberta, Canada, has demonstrated that proteinuria of increasing severity is associated with a faster rate of renal decline regardless of baseline eGFR [51]. A similar main independent prognostic role of proteinuria has been confirmed in the specific setting of tertiary nephrology care [52, 53]. Indeed, the new classification of CKD recently issued by KDIGO highlights the major role of albuminuria [54].

On the other hand, a decrease of proteinuria following therapeutic interventions with anti-RAS agents heralds a better renal prognosis over time in most patients, even in those starting with moderate proteinuria [55–57].

Therefore, albuminuria (or proteinuria) identifies patients at risk for adverse clinical outcomes and efficacious antiproteinuric (antialbuminuric) approaches improve long-term prognosis. Inhibition of the renin-angiotensin system (RAS) certainly is the cornerstone of treatment in proteinuric patients with the effect being largely independent of blood pressure control [58]. However, the high complexity of the system with multiple-level escape mechanisms prevents adequate suppression. Indeed, monotherapy with either angiotensin converting enzyme inhibitor (ACEi) or angiotensin receptor blocker (ARB) decreases proteinuria by not more than 20–30% [59]. Combined use of ACEi and ARB can be an efficacious strategy to further decrease proteinuria especially in nondiabetic CKD [60], but safety issues prevent wider implementation of dual blockade [3, 4].

Novel antiproteinuric strategies aimed at attaining remission of proteinuria (<0.5 g/24 h) are actively being sought. Under this point of view, of great interest are the experimental data linking vitamin D with albuminuria by means of its anti-RAS and anti-inflammatory effects [5, 13, 61].

Recent interventional studies have disclosed that nutritional vitamin D repletion or administration of active vitamin D, that is, a less potent and more calcemic vitamin D analog [62], reduces proteinuria in patients with milder degrees of renal disease, such as microalbuminuric diabetic nephropathy with moderate GFR impairment and IgA nephropathy with close-to-normal GFR [63, 64]. In patients with more advanced disease (CKD stages 3 to 5 and/or macroalbuminuria-proteinuria), consistent data on antiproteinuric effect (−30% on average) have been almost exclusively provided for paricalcitol, an active analogue of vitamin D with low calcemic effect when used at low dose (1 mcg/24 or 48 h), in diabetic as nondiabetic patients with residual proteinuria after anti-RAS therapy [6, 65–69]. In particular in vital study, the authors found that the use of paricalcitol was associated with a reduction of blood pressure, probably for the previously mentioned effect on renin-angiotensin system [68].

These data have been consolidated in two recent large meta-analyses also showing a substantial safety on markers of mineral bone disease (MBD) of this therapy [70, 71].

The effectiveness of paricalcitol in proteinuric CKD, as well as the awareness of albuminuria as determinant of poor renal prognosis in kidney transplant recipients (KTR) [72], has led investigators to test the antialbuminuric property of paricalcitol also in KTR patients. Two studies deserve to be mentioned. The first one was an observational work with long follow-up (up to 24 months) in 58 patients, transplanted by 6 yrs on median, with mean eGFR 35 mL/min/1.73 m^2^ and proteinuria of 1.1 g/24 h [73]. In this study, paricalcitol at the dose of 1 mcg/48 h induced a 36% reduction of proteinuria that was associated with a significantly slower decline of eGFR during the 24 months of treatment as compared to the 24 months before. More recently, the group headed by Remuzzi has completed a 6-month randomized controlled trial showing that oral paricalcitol (1 to 2 mcg/day) in 43 recipients of renal transplants with secondary hyperparathyroidism induced, besides the better control of markers of MBD obtained in the absence of hypercalcemia-phosphatemia, a significant reduction of proteinuria (from 0.27 to 0.14 g/24 h on average) [74].

## 4. Vitamin D and Hypertension

In the last decade, observational or epidemiologic studies consistently indicated that hypovitaminosis D is associated with higher all-cause mortality rates, including those from cardiovascular diseases [75–77]. In particular, the mean serum 25-hydroxyvitamin D levels have been reported to be significantly lower in patients with stable coronary artery disease than in healthy control subjects and independently associated with extent and complexity of coronary artery disease and hypertension [78]. In this regard, previously reported evidence suggested that vitamin D inadequacy may be involved in the development of hypertension. The Third National Health and Nutrition Examination Survey showed that an inverse relationship existed between 25-hydroxyvitamin D and systolic blood pressure, and this relationship remained significant even after adjustment for age, sex, ethnicity, physical activity, and body mass index [79]. Moreover, a retrospective analysis of 2 large cohort studies showed that men whose plasma levels of 25-hydroxyvitamin D were in the lowest (<15 ng/mL) category were at the highest risk of hypertension relative to men whose levels of 25-hydroxyvitamin D were in the highest (≥30 ng/mL) category (relative risk 6.13). In the same comparison with women, the multivariate relative risk was 2.67 [80].

Among the pathophysiological mechanisms, still largely unknown, underlying the association between hypovitaminosis D and hypertension, a key role could be played by vitamin D-mediated suppression of renin biosynthesis through regulation of the renin-angiotensin system (RAS) [37]. Data from animal studies indicated that circulating active vitamin D may act as an inhibitor of renin expression in the juxtaglomerular apparatus and vascular smooth muscle cell proliferation [81]. Vitamin D receptor activation inhibits intrarenal mRNA levels and protein expression of key components of RAS (angiotensinogen, renin, renin receptors, and angiotensin II type 1 receptor) independently of calcium metabolism in mice and rats [39, 40]. Notably, these findings have not been replicated in humans since no suppressive effect on systemic RAS has been found in patients treated with vitamin D [82] and in essential hypertensives after short-term calcitriol administration and after long-term cholecalciferol therapy [83]. However, in both studies, patients were under treatment with RAS inhibitors. Recently, chronic vitamin D receptor stimulation by cholecalciferol therapy has been shown to blunt systemic RAS activity in essential hypertensive patients with hypovitaminosis D under constant salt intake and free from drugs interfering with RAS [84]. Moreover, compared with sufficient vitamin D status, vitamin D deficiency has been associated with a decreased arterial response to angiotensin II challenge (increased delta brachial pulse-wave velocity and delta aortic augmentation index) and increased arterial stiffness in healthy humans, possibly through an angiotensin II-dependent mechanism [85].

Since suboptimal vitamin D levels are linked with development of hypertension, it could be assumed that vitamin D replacement or normalization would reduce the risk of cardiovascular disease and its effects. Results from interventional studies strongly suggested that vitamin D supplementation had a great blood pressure lowering effect and overall improved cardiovascular risk profile [75, 86–88].

The supplementation for 8 weeks with vitamin D plus calcium in 148 vitamin D-deficient elderly women significantly lowered systolic blood pressure by 9.3%, while the calcium-only supplementation lowered it by 4.1% compared to baseline [86]. These results were confirmed by a subsequent double-blind, parallel group, and placebo-controlled randomized trial in vitamin D-deficient type 2 diabetes patients, which were administered with a single dose of 100,000 international units (IU) vitamin D2 or placebo for 8 weeks. Vitamin D supplementation significantly improved flow mediated vasodilatation (FMD) of the brachial artery by 2.3% and decreased systolic blood pressure by 14 mmHg compared with placebo. The improvement in FMD remained significant after adjusting for changes in blood pressure. However, changes in FMD did not correlate with the reduction of systolic blood pressure [87]. A positive correlation between FMD and 25(OH)D was also observed in asymptomatic vitamin D-deficient subjects supplemented with 300,000 IU monthly for 3 months. FMD measurements significantly improved after replacement therapy and resulted significantly lower than controls. Additionally, posttreatment values of lipid peroxidation indexes were significantly lower than pretreatment levels, and a negative correlation between FMD and lipid peroxidation indexes was also observed [88].

Further observations demonstrated that daily supplementation of 2000 IU of vitamin D for 16 weeks optimized vitamin D levels and significantly improved carotid-femoral pulse-wave velocity, a cardiovascular surrogate marker, in 49 young black people with vitamin D insufficiency or deficiency [89].

However, despite the large number of clinical studies carried out to examine the effect of vitamin D supplementation on blood pressure, no univocal data are available on the potential antihypertensive effect of vitamin D. As discussed by a recent meta-analysis on vitamin D supplementation and cardiovascular events, this might be due to heterogeneity of patient baseline characteristics, differences in sample size and follow-up periods, and different vitamin D doses. Indeed, most of randomized controlled trials of vitamin D supplementation and blood pressure mainly have given vitamin D for short periods (<6 months) or at low doses (400 IU per day) [90]. Moreover, the use of different vitamin D formulations produced differences in blood pressure reduction, as shown by ergocalciferol or cholecalciferol with ultraviolet B demonstrating a greater decrease in systolic blood pressure (−6.2 mmHg) than calcitriol (0.7 mmHg) [75]. Notably, recent findings suggest that the association between vitamin D status and elevated blood pressure noted in observational studies may not to be causal. Indeed, vitamin D supplementation did not reduce blood pressure in individuals with pre- or stage I hypertension and vitamin D deficiency [91]. Six months of intermittent, high-dose oral vitamin D3 supplementation did not reduce blood pressure or left ventricular mass in patients with resistant hypertension [92]. Moreover, a long-term (18 months) vitamin D supplementation, increasing the mean 25-hydroxyvitamin D3 concentration >100 nmol/L, had no effect on systolic or diastolic blood pressure in healthy adults without severe vitamin D deficiency [93].

Essential hypertension is a typical example of a complex, multifactorial, and polygenic trait where different metabolic pathways are involved (inflammation, coagulation cascade, sodium reabsorption, cellular adhesion, and lipid metabolism). Some gene variants, contributing to between 30% and 50% of the variation in blood pressure among humans, have been identified so far that interact with environmental factors to produce the hypertensive phenotype. Recently, evidence for an important role of the endothelial vitamin D receptor (VDR) in regulating endothelial function and blood pressure has been provided [94]. Moreover, VDR gene polymorphisms (BsmI, ApaI, and FokI) have been shown to be associated with left ventricle hypertrophy, atherosclerosis, and essential hypertension [95–98].

A decade ago a study investigating the relationship between bone mineral density (BMD) and carotid artery intimal medial thickness (IMT), as a surrogate marker of endothelial dysfunction, among 471 Mexican women, showed that forearm BMD and IMT were significantly higher in individuals having the VDR BsmI BB genotype. Furthermore, the association of the VDR genotype with IMT was not dependent on the association between VDR and BMD [99]. In contrast, no significant difference was detected in biochemical parameters and physical examination between groups for BsmI and ApaI VDR gene polymorphisms in a subsequent study including 74 hypertensive patients (49 females/25 males) without other comorbidities, that is, diabetes mellitus, impaired glucose tolerance, and severe obesity [100]. Interestingly, a negative correlation was observed between vitamin D levels and day-time interval and early morning average blood pressure in the FokI non-FF (Ff/ff, *n* = 35) group compared with the FF one (*n* = 39). Serum cystatin-C was higher in the non-FF group, and the degree and presence of retinopathy were significantly higher in the non-FF group when compared to the FF group [100].

These results were confirmed by a large case-control study investigating the relationship between the VDR FokI polymorphism and essential hypertension in 280 patients and 200 healthy subjects. The risk for hypertension in FF homozygotes was found 2.2 times greater than in Ff heterozygotes and 2.2 times greater than in ff homozygotes, regardless of the presence of family history and smoking status However, when comparing Ff and ff genotypes, no significant difference was observed [101].

Recently, a prospective study has been carried out which aimed to evaluate the association of circulating vitamin D metabolites, VDR FokI and BsmI gene polymorphisms, and their interaction with risk of hypertension [102]. Briefly among the recruited 1,211 US men that were free of baseline hypertension 695 men developed incident hypertension during 15.3-year follow-up. After multivariable adjustment statistical analysis showed that carriers of VDR BsmI bB or BB had a relative risk for hypertension increased by 1.25-fold compared with carriers of bb, while carriers of VDR FokI ff had a relative risk increased by 1.32-fold compared with carriers of FF and Ff combined. Moreover, evidence for an inverse association between plasma 25(OH)D and risk of hypertension was found even if the relation between plasma 25(OH)D and risk of hypertension did not differ by VDR BsmI and FokI polymorphisms [102].

Notably, it has been reported that VDR mutant mice are characterized by lower bioavailability of the vasodilator nitric oxide (NO) due to reduced expression of endothelial nitric oxide synthase, leading to endothelial dysfunction, increased arterial stiffness, increased aortic impedance, structural remodeling of the aorta, and impaired systolic and diastolic heart function at later ages, independent of changes in the renin-angiotensin system [103]. In the light of these reported observations, it may be assumed that more research is needed to further evaluate the role of vitamin D polymorphisms in hypertension development and the usefulness of vitamin D supplementation in hypertension prevention.

## 5. Conclusion

Beyond the traditional involvement in mineral metabolism, vitamin D may interact with other kidney hormones such as renin and erythropoietin. This interaction would be responsible for some of the systemic and renal effects associated with VDR activation. The administration of analogues of vitamin D has been associated with an improvement of anaemia and reduction in ESA requirements [15–17]. The associations found in clinical studies and the supporting mechanistic studies make it plausible that vitamin D deficiency could indeed contribute to an inappropriately elevated renin levels, as a mechanism for progression of CKD and/or cardiovascular disease [5–7]. Consequently the beneficial effects of vitamin D receptor activators in experimental chronic renal failure could be related to downregulation of the renal renin-angiotensin system and in particular to a reduced renin build-up caused by the disruption of the feedback inhibition loop [5–7].

Studies in large patients series and with adequate follow-up are definitely needed to confirm the effects of long-term paricalcitol treatment in CKD and its potential role in improving renal outcome in comparison with not only placebo but also other vitamin D metabolites and analogues [65–74]. Meanwhile, however, it is plausible to suggest that treatment with active vitamin D analogues represents a therapeutic option in proteinuric CKD that can be used in patients intolerant to anti-RAS agents or, as add-on therapy, in those already treated with anti-RAS but not reaching the safe threshold level of proteinuria (<0.5 g/24 h) [69].
